# The silkworm gustatory receptor BmGr63 is dedicated to the detection of isoquercetin in mulberry

**DOI:** 10.1098/rspb.2022.1427

**Published:** 2022-10-26

**Authors:** Shaoyu Zhang, Jiaqi Tang, Yunfeng Li, Dong Li, Guo Chen, Lin Chen, Zhen Yang, Ningjia He

**Affiliations:** State Key Laboratory of Silkworm Genome Biology, Southwest University, Chongqing, People's Republic of China

**Keywords:** feeding mechanism, gustatory receptor, interaction, isoquercetin, oocyte recording

## Abstract

Gustatory systems in phytophagous insects are used to perceive feeding stimulants and deterrents, and are involved in insect decisions to feed on particular plants. During the process, gustatory receptors (Grs) can recognize diverse phytochemicals and provide a molecular basis for taste perception. The silkworm, as a representative Lepidoptera species, has developed a strong feeding preference for mulberry leaves. The mulberry-derived flavonoid glycoside, isoquercetin, is required to induce feeding behaviours. However, the corresponding Grs for isoquercetin and underlying molecular mechanisms remain unclear. In this study, we used molecular methods, voltage clamp recordings and feeding assays to identify silkworm BmGr63, which was tuned to isoquercetin. The use of qRT-PCR confirmed that *BmGr63* was highly expressed in the mouthpart of fourth and fifth instar larvae. Functional analysis showed that oocytes expressing *BmGr63* from the ‘bitter’ clade responded to mulberry extracts. Among 20 test chemicals, BmGr63 specifically recognized isoquercetin. The preference for isoquercetin was not observed in *BmGr63* knock-down groups. The tuning between BmGr63 and isoquercetin has been demonstrated, which is meaningful to explain the silkworm-mulberry feeding mechanism from molecular levels and thus provides evidence for further feeding relationship studies between phytophagous insects and host plants.

## 1. Introduction

Relationships between phytophagous insects and their host plants are complex and have developed over long-term coevolution [1]. Plants provide food for insects, while plants with different characteristics can significantly affect insect growth, development and geographical distribution [2,3]. Phytophagous insects have developed specific feeding preferences and food adaptation strategies for different plant species [4]. In seeking food, insect chemosensory systems (including olfactory and gustatory systems) are used to detect plant-based chemical signals [5–8]. When an insect starts to feed, gustatory sensilla mainly on the mouthparts make direct contact with plant sap released by chewing [9]. Gustatory systems recognize non-volatile chemicals and are indispensable for the integrated assessment of feeding stimulants and deterrents in plants [5,10,11]. When non-volatile chemicals enter the gustatory sensilla, gustatory receptors (Grs) located on the gustatory receptor neurons will recognize and resolve the chemicals and convert chemical signals to electrical signals. These signals are then transmitted to the central nervous system and guide feeding behaviour [11,12]. Gustatory system evolution facilitates insect adaptation to different ecological niches [13,14].

The first insect Grs were identified in *Drosophila melanogaster* [15,16]. Additional Gr data are limited to published genome sequences of particular species including *Bombyx mori* [17–19], *Heliconius melpomene* [20], *Plutella xylostella* [21–23] and *Manduca sexta* [24]. Insect Grs often possess seven transmembrane domains with reverse topology when compared with classical G-protein coupled receptors, with an intracellular N-terminus and an extracellular C-terminus [16,18]. Grs are diverse, with low sequence similarities of 15–25% [16], and classification into four clades including the CO_2_, fructose/inositol, sugar and bitter clades. Sugar Grs have been functionally characterized in several insect species. For example, Gr43a in *D. melanogaster* responds to fructose [25] In *P. xylostella*, two spliced variants of PxylGr43a can respond to fructose [26]. Bitter receptor function is relatively unknown and has been mainly studied in *D. melanogaster*. DmGr28b is necessary for saponin avoidance [27]. The subsets of DmGr66a and some other Grs in *Drosophila* may be responsible for detecting bitter compounds. For example, Gr33a, Gr66a and Gr93a are all required to generate a functional umbelliferone receptor [28]. Recent studies reported bitter receptor function in Lepidoptera. In *Papilio xuthus*, PxutGr1 is specific for the oviposition stimulant synephrine and is a key factor in host specialization [29]. PxylGr34 from *P. xylostella* is responsible for the steroid plant hormone brassinolide as a feeding and oviposition deterrent [30]. PrapGr28 in *Pieris rapae* is tuned to sinigrin, a stimulant for larval feeding and adult oviposition [31]. However, many insect *Gr*s have not been functionally characterized. The identification of their ligands should help elucidate insect adaption to different ecological niches and their host plant specificity.

Silkworm larvae predominantly feed on mulberry leaves. Mulberry is widely planted in the Eurasian continent and is important for domesticated silkworm culture [32]. The silkworm mulberry feeding mechanism has been studied as a representative example of interactions between a phytophagous insect and its host plants. Hamamura *et al*. [33–35] proposed that there were attraction, biting and swallowing factors in mulberry leaves, which stimulated silkworm feeding, and relatively few avoidance factors inhibited feeding, thereby triggering larval feeding behaviour. For instance, volatile components in mulberry such as cis-jasmone are highly attractive to silkworm larvae and are recognized by olfactory systems [36]. Non-volatile compounds such as polysaccharides, β-sitosterol and isoquercetin act as gustatory factors to promote silkworm feeding [34,37]. Previous studies focused on the direct effects of phytochemicals on silkworm feeding behaviour, but the molecular mechanisms underlying feeding behaviour were not fully understood. With the development of genome and transcriptome sequencing, *Gr*s in *B. mori* have been identified, further re-annotated and now number 76 [17,19]. In sugar clades, BmGr9 responds to fructose [38] and BmGr8 and BmGr10 to inositol [18,39]. In bitter clades, BmGr16, 18 and 53 broadly respond to the feeding deterrents coumarin and caffeine [40]. Knock-out of BmGr66 increases the plants accepted by silkworm larvae in addition to mulberry leaves, but potential *BmGr66* ligands remain to be identified [41]. Mulberry-derived ligands corresponding to specific BmGrs also remain unknown. Metabolome sequencing has successfully characterized many mulberry metabolites [42]. Identification of molecular interactions between BmGrs and mulberry phytochemicals will help reveal the silkworm mulberry feeding mechanism and provide a reference for other studies of relationships between phytophagous insects and their host plants.

In the present study, multi-level experiments on the interaction between BmGrs and mulberry metabolites were conducted. *BmGr*s expressed in larval mouthparts, based on previous transcriptome data, were selected and full-length coding sequences were identified. Then, candidate *BmGr*s expression levels in larval mouthparts were determined using quantitative real-time PCR (qRT-PCR). *BmGr63*, with the highest expression levels, was subjected to ligand identification in voltage clamp experiments. Furthermore, RNA interference (RNAi) methods were adopted to demonstrate interactions between BmGr63 and ligand in silkworms.

## 2. Material and methods

### (a) Animal rearing and plants

The DAZAO silkworm strain was used in all the experiments. It was maintained at the Gene Resource Bank of Domesticated Silkworms (Southwest University, Chongqing, China). Silkworm larvae were reared at 25°C under a 12 : 12 h (light : dark) photoperiod and fed mulberry leaves or artificial diets (purchased from Shandong Agricultural University) in feeding assays. One-day-old fourth and fifth instar larval mouthparts were collected to examine candidate *BmGr*s expression levels. Also, 2-day and 3-day-old fifth instar larval labrum, maxilla, labium, mandible, thoracic leg and midgut were collected to study *BmGr63* expression patterns. Collected tissues were washed in phosphate buffered saline (PBS) buffer and stored at −80°C until use.

Female *Xenopus laevis* were purchased from Haiwei Panshi Biomedical Technology Co., Qingdao, China, and reared on frog diets at 18–20°C in our laboratory. Healthy female *X. laevis* individuals were anaesthetized on ice for 30 min before surgically collecting the oocytes.

The mulberry leaves of *M. alba var. Shin-Ichinose* (a cultivated resource) were harvested from the Mulberry Germplasm Nursery (Southwest University, Chongqing, China). To extract total active substances, freeze-dried mulberry leaf powder was soaked in 40% methanol (1 : 10, m/v), and vortexed three times with 10 min intervals. The solution was then placed at 4°C overnight. For oocyte experiments, the filtered extract solution was concentrated and then diluted to a working concentration in Ringer's solution.

### (b) RNA extraction and qRT-PCR analysis

Total RNA was extracted from collected tissues using an RNAiso Plus Kit (Takara Bio., Shiga, Japan). Total RNA (1 µg) was used as the template to synthesize cDNA with the PrimeScript RT Reagent Kit (Perfect Real Time) (Takara Bio., Shiga, Japan). Then, qRT-PCR assays were conducted using the QuantiNova SYBR Green PCR Kit (QIAGEN, Hilden, Germany). *Glyceraldehyde-3-phosphate dehydrogenase* (*GAPDH*) served as a reference control for normalizing CT values. Gene-specific qRT-PCR primers were designed using Primer Premier 5 (electronic supplementary material, table S1). The 2^−ΔΔCT^ method was used to calculate the relative fold changes in gene expression [43]. The qRT-PCR was performed in three independent technical replicates.

### (c) Cloning of *BmGr*s and vector construction

We referred to published transcriptome data [19] to screen for *Gr*s expressed in larval mouthparts. As these predicted *BmGr*s sequences were incomplete or inaccurate, specific primers were designed to obtain full-length coding sequences. PCR was conducted using Platinum SuperFi II Green PCR Master Mix (Invitrogen, Carlsbad, CA, USA) as follows: 98°C for 30 s, followed by 35–40 cycles of 98°C for 10 s, 60°C for 10 s and 72°C for 1 min, and a final extension at 72°C for 5 min. Amplified *BmGr*s were then cloned into a pEASY-Blunt vector (TransGen Biotech, Beijing, China) and sequenced. *Gr*s that terminated early or were un-cloned were removed and the rest were used as candidate *Gr*s for follow-up experiments (electronic supplementary material, table S2).

To confirm BmGr63 localization in human embryonic kidney 293T (HEK293T) cells, an mScarlet tag with red fluorescence was fused with *BmGr63* into the pcDNA3.1 vector. The full-length *BmGr63* coding sequence was inserted between *Bgl*II and *Bcu*I sites in the pT7Ts expression vector and used for cRNA synthesis.

### (d) Phylogenetic analysis and transmembrane domain prediction

To investigate evolutionary relationships between BmGr63 and Grs from *B. mori*, and other Lepidoptera species, a phylogenetic tree based on Gr protein sequences from *B. mori*, *M. sexta*, *H. melpomene*, *P. xylostella* and *P. rapae* was constructed (electronic supplementary material, table S3). Amino acid sequences were aligned using MAFFT v.7.455 [44], and gap sites were removed with trimAl v.1.4 [45]. The maximum likelihood phylogenetic tree was constructed using RAxML v.8.2.12 [46] with the Jones–Taylor–Thornton amino acid substitution model with a bootstrap of 5000 replicates. The phylogenetic tree was constructed with iTOL (https://itol.embl.de). As the topology was required for receptor function, BmGr63 transmembrane domain predictions were performed using HMMTOP (http://www.enzim.hu/hmmtop/).

### (e) Cell culture and transfection

Cells were cultured in Dulbecco's modified eagle medium (DMEM) (HyClone, Chicago, IL, USA) containing 10% fetal bovine serum (FBS) (Gemini, West Sacramento, CA, USA) and 1% penicillin and streptomycin (Thermo Scientific, Waltham, MA, USA) at 37°C with 5% CO_2_. The *BmGr63* recombinant vector was transfected into HEK293T cells using liposomal polyethyleneimine (PEI) (Invitrogen, Carlsbad, CA, USA). Cells were seeded on round coverslips for 24 h until adherence. Then DNA and PEI were mixed in a ratio of 1 : 2 in 0.4 ml optimized-minimal essential medium (OPTI-MEM) for 20 min and added to wells. After 4 h, the medium was replaced by fresh OPTI-MEM medium containing 10% FBS 4 h later. At 24 h after transfection, red fluorescence was observed using a ZEISS LSM880 system equipped with a 63× oil objective (NA 1.4) and controlled by ZEN 2.1 software at an excitation wavelength of 488 nm. Acquired images were analysed using ImageJ software.

### (f) Two-electrode voltage clamp recordings

The cRNA was synthesized from the linearized pT7Ts vector using *Bam*HI with mMESSAGE mMACHINE T7 Kit (Ambion, Austin, TX, USA). Purified cRNA was diluted to 2 µg µl^−1^ and stored at −80°C until use. Surgically collected oocytes were treated with 2 mg ml^−1^ collagenase type I (Sigma-Aldrich, St. Louis, MO, USA) in Ca^2+^ free buffer (96 mM NaCl, 2 mM KCl, 1 mM MgCl_2_, 5 mM HEPES, pH = 7.6) for 1–2 h at room temperature. Mature oocytes were microinjected with 25 nl *BmGr63* cRNA, and RNase-free water as a negative control. Injected oocytes were incubated for 3–5 days at 18°C in Barth solution (88 mM NaCl, 1 mM KCl, 1 mM MgCl_2_, 0.4 mM CaCl_2_, 0.3 mM Ca(NO3)_2_, 1 mM MgSO_4_, 2.4 mM NaHCO_3_, 10 mM HEPES, pH = 7.4) supplemented with 5% dialyzed horse serum, 50 mg ml^−1^ penicillin, 50 mg ml^−1^ streptomycin, 100 mg ml^−1^ gentamycin and 550 mg ml^−1^ sodium pyruvate. The two-electrode voltage clamp technique was used to record whole-cell currents in oocytes responding to mulberry extracts and chemical compounds. Intracellular glass electrodes were filled with 3 M KCl and presented resistances of 0.3–3.0 MΩ. Signals were amplified with an OC-725C amplifier (Warner Instruments, Hamden, CT, USA) at a holding potential of −80 mV, low-pass filtered at 50 Hz and digitized at 1 kHz. When the baseline was stable, compounds for testing were perfused and current changes were recorded. Then oocytes were washed with Ringer's solution until the current returned to a stable baseline. Test compounds were changed using a peristaltic pump (BT100-2 J, Longer Precision Pump Co., Ltd, Baoding, China) at a constant speed. Mulberry extracts at different concentrations were used to stimulate oocytes. Then the listed chemical compounds (electronic supplementary material, table S4) in 1.0 mM in Ringer's solution (96 mM NaCl, 2 mM KCl, 0.1 mM CaCl_2_, 2.0 mM MgCl_2_, 5 mM HEPES, pH = 7.6) were used to record currents. When a clear current response was observed at 1 mM, compounds at gradient concentrations were tested as described.

### (g) RNAi mediated by double-stranded RNA (dsRNA)

The dsRNA interfered region was designed using DRSC (https://www.flyrnai.org/cgi-bin/RNAi_find_primers.pl). A verified *BmGr63* sequence template was amplified with specific primers (shown in electronic supplementary material, table S1) containing the T7 promoter to acquire PCR products for the interfered region. PCR products were then used to yield dsRNA using a T7 High Efficiency Transcription Kit (TransGen Biotech, Beijing, China) following protocols. Green fluorescent protein (GFP) dsRNA was synthesized using the same method as the control. The obtained dsRNA was stored at −80°C until use.

For fourth instar larvae, 3 µg dsRNA was added to artificial diets and used to feed silkworms. The larvae that consumed the given diets were starved for another 12 h and then used in RNAi experiments. For fifth instar larvae, 5 µg dsRNA was brushed on mulberry leaves and used to feed silkworms. The larvae that consumed the entire leaves were starved for another 12 h, and used in RNAi experiments.

### (h) Feeding assays

Artificial diet was used to examine isoquercetin effects on silkworm feeding. Freshly moulted fourth instar larvae were starved for 1 day before experiments. The dry diet weight to solution ratio was 1 : 1.5. Test compound solutions, in gradient concentrations, were used in experimental groups. For control groups, water was used. Larvae were placed separately into Petri dishes and the experiments were performed at 25°C. Consumed diet weight was calculated using the difference in diet wet weight before testing and at 3 h after testing.

In RNAi experiments, no choice and dual choice feeding assays were performed. Silkworms were separated into *BmGr63* RNAi and control groups. In no choice feeding assays, artificial diets adding 10^−3^ M isoquercetin were used. In dual choice feeding assays, artificial diets with or without the addition of isoquercetin were used. The method was described above.

We investigated *BmGr63* function in silkworm feeding. Freshly moulted fifth instar larvae, starved for 1 day, were used. Silkworms of uniform size were used for further assays. Three larvae were used in a group and the number of biological replicates was five groups. A similar weight of mulberry leaves was used in each group, and the weight of eaten leaves was calculated by differences in weight before and at 2 h after testing.

### (i) UPLC-MS/MS system and analytical conditions

Mulberry extracts were obtained using aforementioned methods. An isoquercetin standard was diluted to different concentrations (10^−3^ M, 5 × 10^−3^ M and 10^−2^ M). Then the extracts and standard solutions were analysed using an LC-ESI-MS/MS system (Thermo Fisher Scientific, Waltham, MA, USA). The ultra-high performance liquid chromatography (UPLC) conditions were previously reported [42]. Relative isoquercetin content (peak area) was calculated using Thermo Scientific Xcalibur software (v.2.2) with accurate identification and rational detection parameters: mass tolerance (units), 10 ppm; mass precision, 0.0001; retention time (min), precision, 0.01; smoothing points, 10; baseline window, 50; area noise factor, 5; peak noise factor, 10.

## 3. Results

### (a) *Bmgrs* expression levels in silkworm larval mouthparts

**Figure 1.**
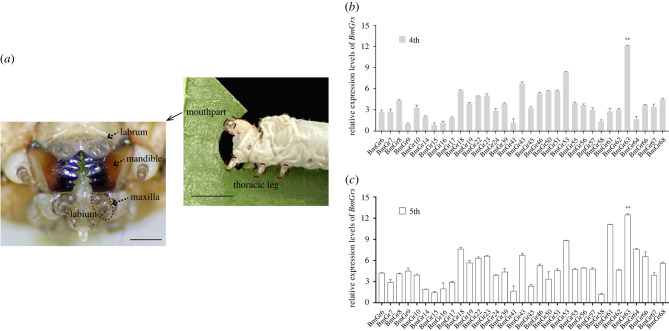
Relative expression levels of *BmGr*s in larval mouthparts. (*a*) Feeding behaviour of fifth instar silkworm larvae. In the upper image, mouthpart and thoracic leg are indicated (the scale bar represents 5 mm). In the lower image, the mouthpart is enlarged and labrum, mandible, labium, maxilla are indicated (the scale bar represents 250 µm). (*b*,*c*) The relative expression levels in fourth and fifth instar mouthpart were determined using qRT-PCR assays and calculated based on the 2^−ΔΔCt^ method. The *BmGAPDH* gene was used as the internal control to normalize expression levels. The fold change of relative expression levels was used to display the expression pattern. Data are presented as the mean ± s.e.m. of three replicates and statistically analysed using a least-significant difference (LSD) test (the significance analysis about expression levels of candidate *BmGr*s was shown in electronic supplementary material, table S6). (Online version in colour.)

The cloned sequences of *BmGrs* expressed in mouthparts without early termination are listed in electronic supplementary material, table S2. Although RNA-seq reflects overall trends in gene expression, qRT-PCR validation is required to verify the expression of important genes. Thus, qRT-PCR was conducted to determine the spatial-temporal expression levels of candidate *BmGrs*. And it showed that *BmGr63* was the most highly expressed *Gr* in fourth and fifth instar larval mouthparts (figure 1).

### (b) Phylogenetic analysis

**Figure 2.**
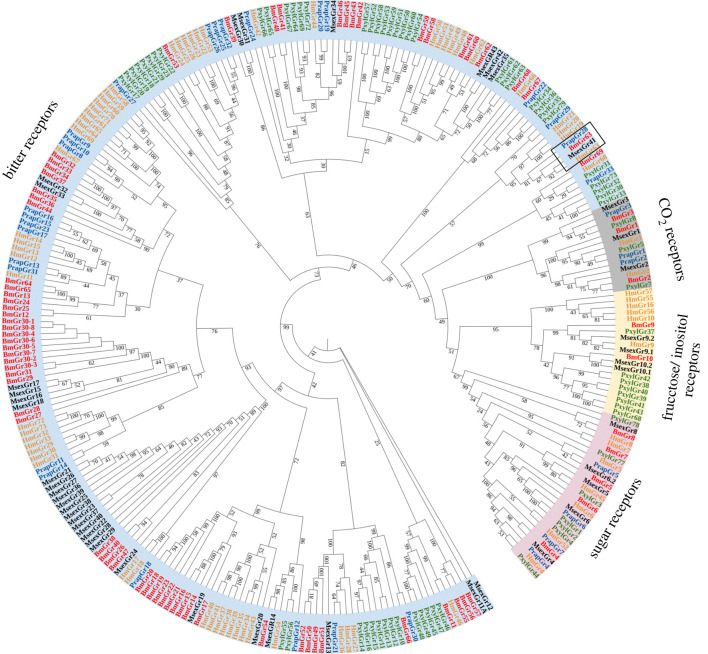
Phylogenetic relationships among gustatory receptor proteins from Lepidoptera. The sequence information used is given in electronic supplementary material, table S3. The insect species used in the phylogenetic analysis: Bm, *Bombyx mori* (red); Msex, *Manduca sexta* (black); Hm, *Heliconius melpomene* (orange); Pxyl, *Plutella xylostella* (green); Prap, *Pieris rapae* (blue). The *BmGr63* subclade was marked with a box. (Online version in colour.)

As shown in figure 2, the phylogenetic tree was divided into CO_2_, fructose/inositol, sugar and bitter clades. BmGr1–3 were clustered in the CO_2_ clade, BmGr4–8 belonged to the sugar clade and BmGr9–10 were in the fructose/inositol clade. The remaining 66 BmGrs, were divided into bitter clades with uncharacterized functions. The BmGr63 subclade included PrapGr28, MsexGr41 and HmGr63.

### (c) Bmgr63 structural and expression analysis

**Figure 3.**
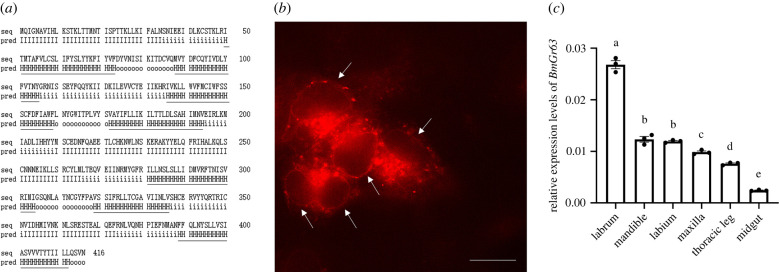
Structural analysis, cellular localization and expression profiles of BmGr63. (*a*) The prediction of transmembrane domains using HMMTOP. ‘H’ stands for the transmembrane domain, ‘i’ stands for the region inside the membrane, and ‘o’ stands for outside. (*b*) Image of HEK293T cells expressing BmGr63 fused with mScarlet. The arrows indicate signals located on membranes. The scale bar represents 10 µm. (*c*) Relative expression levels of *BmGr63* in labrum, mandible, labium, maxilla, thoracic leg and midgut were based on qRT-PCR assays and calculated using the 2^−ΔΔCt^ method. *BmGAPDH* was used as a reference gene. Data are presented as the mean ± s.e.m. of three replicates. Different letters above bars represent significant differences (*p* < 0.05) as determined with the ANOVA one-way Duncan's multiple range test. (Online version in colour.)

Full-length *BmGr63* coding sequences were obtained through gene cloning and the open reading frame encoded a 417 amino acid protein (figure 3*a*). Using HMMTOP analysis, it was predicted that BmGr63 had seven transmembrane domains and the N-terminus was intracellular (figure 3*a*). It should be pointed out that the prediction using different tools might be different (electronic supplementary material, table S2). The combination of multiple prediction methods and experimental verification should be comprehensive. Cellular localization showed that BmGr63 was localized on cell membranes, consistent with Grs functioning as membrane proteins (figure 3*b*).

*BmGr63* expression profiles in specific mouthpart tissues (including labrum, mandible, labium and maxilla), thoracic leg and midgut were also investigated (figure 3*c*). The results indicated that the highest *BmGr63* expression levels were observed in labrum. *BmGr63* was also expressed at relatively high levels in mandible, labium, maxilla and thoracic leg, but was repressed in midgut.

### (d) Identification of interaction between mulberry extracts and *BmGr63* using the oocyte expression system

**Figure 4.**
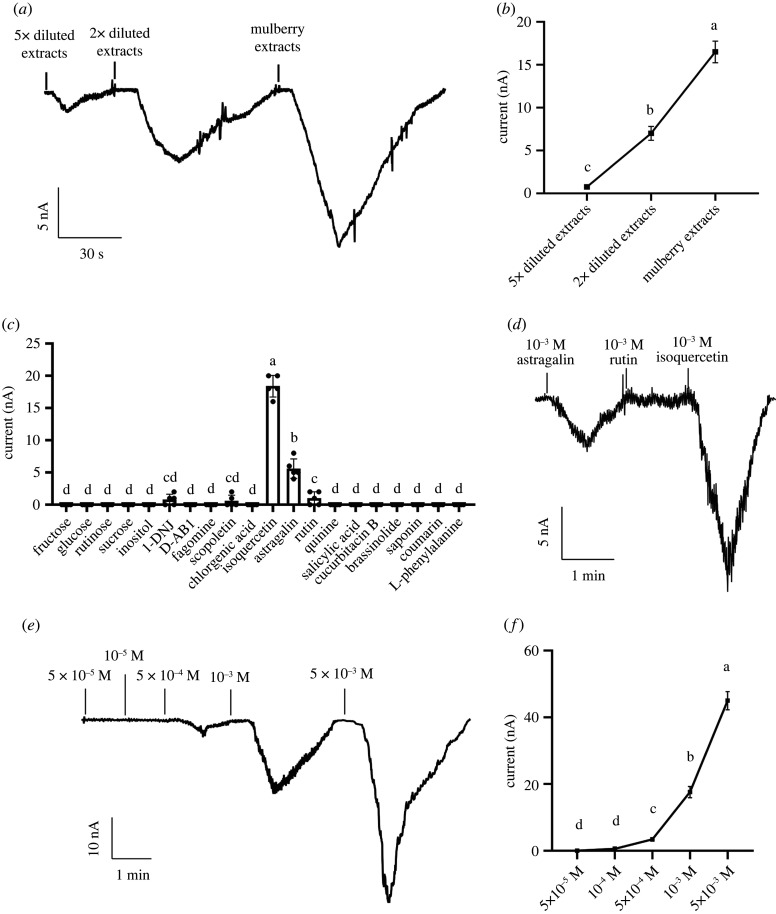
Inward currents recording of oocytes expressing *BmGr63*. The inward current (*a*) and response profiles (*b*) of oocytes expressing *BmGr63* in response to mulberry extracts at different concentrations. (*c*) Response profiles of oocytes expressing *BmGr63* in response to different ligands at 10^−3^ M. (*d*) Inward current responses of oocytes expressing *BmGr63* to different flavonoids. (*e*) Inward current responses of oocytes expressing *BmGr63* to isoquercetin at a range of concentrations. (*f*) Response profiles of oocytes expressing *BmGr63* in response to isoquercetin at a range of concentrations. Data are presented as the mean ± s.e.m. (*n* = 5). Different letters above bars represent significant differences (*p* < 0.05) as determined by the ANOVA one-way Duncan's multiple range test.

To determine whether BmGr63 was involved in silkworm recognition of mulberry leaves, mulberry extracts were used as stimulants for oocytes expressing *BmGr63*. In oocytes, currents induced by mulberry extracts were increased in a dose-dependent manner (figure 4*a*,*b*). Oocytes injected with RNase-free water were used as negative controls and no clear currents were observed at any concentration (electronic supplementary material, figure S1).

### (e) Identification of *BmGr63* ligand with the *Xenopus* oocyte expression system

Voltage clamp recordings were used to clarify which mulberry metabolite class functioned as ligands for BmGr63. Twenty metabolites, at initial concentrations of 10^−3^ M, included sugars, flavonoids, alkaloids, amino acids, phenylpropanoids and plant hormones (electronic supplementary material, table S4). Of the three flavonoid glycosides, figure 4*c*,*d* showed that isoquercetin induced a strong response in the oocytes expressing *BmGr63*. The structural analogue astragalin induced a weaker response while the response to rutin was very weak. A range of isoquercetin concentrations was tested. Currents induced by isoquercetin increased from the lowest threshold concentration of 10^–4^ M in a dose-dependent manner (figure 4*e*,*f*). At 10^−3^ M and 5 × 10^−3^ Mv doses, strong inward currents were observed. Water-injected oocytes as negative controls did not respond to test chemicals (electronic supplementary material, figure S1).

### (f) The effects of isoquercetin on silkworm feeding

**Figure 5.**
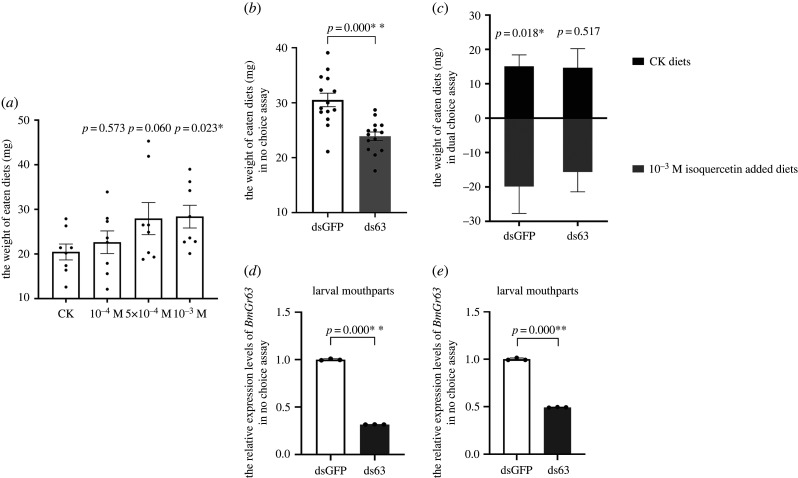
Feeding assays determining the effect of isoquercetin and *BmGr63*. (*a*) Effect of isoquercetin as a feeding stimulant on silkworm larvae. The concentration of isoquercetin added to the artificial diet was 10^−4^ M, 5 × 10^−4^ M, 10^−3^ M, and the artificial diets without treatments were used for control. Data are presented as the mean ± s.e.m. (*n* = 8) and statistically analysed using an LSD test. (*b*) In the no choice assay, artificial diets with 10^−3^ M isoquercetin added were provided. Data are presented as the mean ± s.e.m. (*n* = 14) and analysed for statistical significance using an LSD test. (*c*) In the dual choice assay, artificial diets made by adding 10^−3^ M or without isoquercetin were provided in both dsGFP and ds63 groups. Data are presented as the mean ± s.e.m. (*n* = 12) and analysed for statistical significance using a paired samples *t*-test. (*d*,*e*) Relative expression levels of *BmGr63* were determined using qRT-PCR. Data are presented as the mean ± s.e.m. and analysed for statistical significance using an LSD test.

To study the stimulating or deterrent effects of isoquercetin on silkworm feeding, diet assays were conducted. Food intake increased as isoquercetin concentrations increased and this increase was significant when diets were supplemented with 10^−3^ M isoquercetin (figure 5*a*). Thus, isoquercetin functioned as a feeding stimulant for larval silkworms.

To determine if BmGr63 was involved in isoquercetin recognition in silkworms, RNAi knock-down mediated by dsRNA was used. In no choice assays, when compared with GFP dsRNA-treated larvae, *BmGr63* dsRNA-treated larvae showed a reduced diet intake containing isoquercetin (figure 5*b*). In dual choice assays, GFP dsRNA-treated larvae preferred diets supplemented with isoquercetin, while *BmGr63* dsRNA-treated larvae showed no obvious preference for diets adding isoquercetin (figure 5*c*). *BmGr63* expression levels in larval mouthparts were determined using qRT-PCR to verify RNAi knock-down effects. As shown in figure 5*d*,*e*, *BmGr63* expression levels were downregulated by 50–70% in *BmGr63* dsRNA-treated larvae when compared with GFP dsRNA-treated larvae.

### (g) The influence of *BmGr63* on silkworm food intake

**Figure 6.**
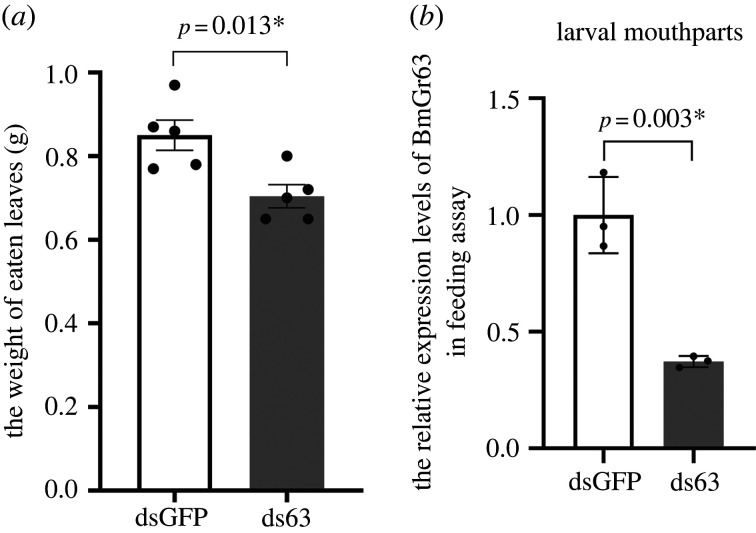
Functional analysis of BmGr63 in food intake of fifth instar silkworm larvae. The fresh mulberry leaves were provided in GFP dsRNA-treated and *BmGr63* dsRNA-treated groups. (*a*) The weight of eaten leaves. (*b*) The larval mouthparts used in the assays were collected and three technical replicates were conducted. The relative expression levels of *BmGr63* were determined using qRT-PCR. Data are presented as the mean ± s.e.m. and analysed for statistical significance using an LSD test.

To determine whether BmGr63 affected food intake, feeding assays were performed. When compared with GFP dsRNA-treated larvae, *BmGr63* knock-down significantly decreased food consumption in silkworm larvae (figure 6*a*). The qRT-PCR analysis confirmed that *BmGr63* levels were reduced by approximately 60% in *BmGr63* dsRNA-treated larvae (figure 6*b*).

### (h) Isoquercetin content analysis in mulberry

Isoquercetin was detected in mulberry extracts using an isoquercetin standard as reference (electronic supplementary material, figure S2). The MS/MS spectra of the standard and isoquercetin in mulberry extracts were identical. A regression line based on the isoquercetin standard peak area was obtained. The equation was y=7E+08x−1E+09, $R^{2}=0.9241$. Accordingly, the isoquercetin concentration in used mulberry extracts was 1.437 × 10^−3^ M.

According to the mulberry leaf metabolite database, isoquercetin content was determined in 91 mulberry resources, with differences identified across resources (electronic supplementary material, figure S3). Resources with higher isoquercetin content were mainly identified in cultivated resources except for ml053. By contrast, wild resources accounted for lower isoquercetin content (electronic supplementary material, table S5).

## 4. Discussion

Mechanisms used by silkworms to feed on mulberry leaves are relevant to relationships between other phytophagous insects and host plants. The mulberry-derived flavonoid glycoside, isoquercetin, was previously found to trigger test biting in silkworm larvae [33–35]. Larval maxillary palps can respond to very low concentrations of isoquercetin [37]. However, the molecular mechanisms underlying host plant recognition remain unknown. From a molecular perspective, contact chemosensation with phytochemicals and ligand binding occurs via Grs [9]. We confirmed that, at the molecular level, a Gr highly expressed in silkworm larval mouthparts, BmGr63, was tuned with the feeding stimulant isoquercetin in mulberry.

### (a) The biological function of *BmGr63* in food selection

When insects are close to food sources, their gustatory systems detect and evaluate non-volatile feeding stimulants or deterrents and are important in the final decision to accept the food [9,47]. Lepidoptera gustatory sensilla are mainly located on maxilla and epipharynx (inner side of labrum) [48–50]. Although important roles for labellum have been demonstrated in *Drosophila*, gustatory perception research in Lepidoptera labrum or epipharynx is lacking [12,51,52]. The sensilla located on maxilla have received more attention. The gustatory sensilla consists of medium and lateral sensillum styloconica in the maxillary pulps and galea and are important in larval food selection [11]. The medial styloconic sensilla of the maxillary galea respond to feeding deterrents or toxic compounds such as coumarin, caffeine and nicotine [53]. Chemosensory neurons in the maxillary palps are tuned with several mulberry compounds (including β-sitosterol, CGA and quercetin glycosides) and are important in host plant selection [37]. However, the underlying molecular mechanisms were unknown. BmGr66 was reported as a determining factor for silkworm feeding preference. And it had the highest expression levels, specifically in maxilla, among mouthpart tissues [41]. It was plausible that BmGr66 on the gustatory sensilla of the maxilla, was part of a feeding deterrent recognition system. Without BmGr66, silkworms demonstrated non-specific feeding behaviours but retained to feed on mulberry leaves. This observation suggested that other Grs recognized feeding stimulants in plants. *BmGr63* is highly expressed in the maxilla palps [19]. Our qRT-PCR data indicated that *BmGr63* was relatively highly expressed in labrum and maxilla. Besides it, BmGr63 was specific for the feeding stimulant isoquercetin and had a positive influence on silkworm feeding. Therefore, BmGr63 appears to promote silkworm feeding on mulberry leaves and is a critical gustatory factor in feeding mechanisms.

It was also observed that *BmGr63* was highly expressed in chemosensory tissues in larvae and adults [19], which further suggested *BmGr63* had broader biological functions. Apart from host plant recognition in larvae, BmGr63 may also be potentially involved in mating behaviours or oviposition.

### (b) Isoquercetin, a flavonoid glycoside, functions as a silkworm feeding stimulant

Mulberry leaves are rich in diverse flavonoids, with flavonoid skeletons typically modified by sugars or other groups [54,55]. The most abundant mono- and di-*O*-glycosylated flavonoids in mulberry leaves are isoquercetin, astragalin and rutin [56]. Silkworms have adapted to these abundant flavonoid glycosides and use them for developmental purposes. For example, dietary flavonoid glycosides are metabolized in silkworms using quercetin 5-*O*-glucosyltransferase to generate quercetin 5-*O*-glucosides, the major constituent of cocoon flavonoids with ultraviolet shielding properties [57]. This is direct evidence for silkworms using flavonoids derived from mulberry leaves. In our study, a mulberry-derived flavonoid glycoside was shown to function as a feeding stimulant that is detected by BmGr63.

Of the three flavonoid glycosides tested, isoquercetin induced the strongest inward current in oocytes expressing *BmGr63*, followed by astragalin. However, rutin induced little response. In structural terms, isoquercetin and astragalin are mono-*O*-glycosylated flavonoids and their glycosyl ligands are glucoses. Rutin is the product of flavonoid disaccharide modification, and its glycosyl ligand is rutinose. Thus, it appears that interactions between BmGr63 and flavonoid glycosides are based on ligand structures. The current induced by undiluted mulberry extracts was 14–20 nA, and that induced by 10^−3^ M isoquercetin was 16–22 nA. The isoquercetin content in mulberry extracts was determined at 1.437 × 10^−3^ M using UPLC-MS/MS. Mulberry extracts comprise multiple compounds with complex inter-relationships. However, using standards was more direct and effective. Evaluated in this manner, it is hypothesized that isoquercetin is the primary factor inducing BmGr63 responses to mulberry extracts. However, the compound types used in our assays were limited and other potential BmGr63 ligands cannot be ruled out. As receptors other than BmGr63 were not tested in oocyte and RNAi experiments, it remains to be studied whether other receptors are involved in recognizing isoquercetin.

Isoquercetin content was relatively higher in cultivated mulberry resources but lower in wild resources (electronic supplementary material, figure S3 and table S5). The feeding tendency of silkworms to wild mulberry resources, typically *Morus notabilis*, was significantly weaker when compared with cultivated resources [32,58]. Different isoquercetin levels in wild and cultivated resources might explain this finding.

### (c) The relationship between phytochemicals and *Gr*s, and associated behaviours

The majority of *Gr*s are classified into the ‘bitter receptor’ clade with unknown functions. Bitter receptors may recognize bitter compounds that are detrimental or toxic to insects and thus elicit avoidance behaviours. For example, Gr28b is necessary for *D. melanogaster* to avoid saponin. PxylGr34 responds to the plant hormones BL and EBL, which are repellant to *P. xylostella*. However, bitter receptors could also be activated by feeding or oviposition stimulants. PxutGr1 from *P. xuthus* is involved in the recognition of the oviposition stimulant, synephrine. Additionally, PrapGr28 responds to sinigrin, a potent stimulant of larval feeding and adult oviposition in *P. rapae*. In this study, we identified the specific interaction between BmGr63 and isoquercetin, a bitter silkworm Gr and a mulberry-derived feeding stimulant.

In the constructed phylogenetic tree, relatively few orthologous relationships were observed, taking BmGr63 as an example, and it was uncommonly orthologous to PrapGr28. The sequence alignment between BmGr63 and PrapGr28 was shown in electronic supplementary material, figure S4. PrapGr28 is abundantly expressed in larval mouthparts and tuned to sinigrin, a potent stimulant of larval feeding and adult oviposition. Sinigrin is a glucosinolate, an important secondary compound in Cruciferae and its glycosyl ligand is glucose [59,60]. From this perspective, the conserved functions of BmGr63 and PrapGr28 are related to the detection of glucose-ligand glycosides. The important roles of plant-derived glycosides in Lepidoptera feeding behaviours are implied.

The ability to recognize secondary metabolites is essential for phytophagous insects. Phytochemicals may not only act as deterrents but, depending on insect ability to circumvent their detrimental effects, they could also act as stimulants and host plant indicators [47]. For insects, the stimulants are recognized during contact chemosensation and received by Grs. Then the produced stimulant signals are transferred to the brain via stimulus neurons, the electrophysiological activity of which correlates quantitatively to the strength of the behavioural response [11,61].

This study showed that BmGr63 has the highest expression levels in larval mouthparts and is specific to the mulberry-derived feeding stimulant isoquercetin. This knowledge provides a molecular basis for silkworm mulberry feeding mechanisms. In future work, comprehensive studies about Grs in Lepidoptera should be made using the combination of electrophysiology, transgenic technologies and behavioural experiments. Also, the effects of phytochemicals on insects, including interactions and transport mechanisms need further elucidation. Our interaction data between BmGrs and mulberry-derived secondary metabolites should help facilitate understanding of the specific evolutionary relationships between phytophagous insects and their host plants.

## Data Availability

All data are available within the manuscript and the electronic supplementary material [62].
